# Supplementary material for: Multiwalled Carbon Nanotubes Induce Fibrosis and Telomere Length Alterations
Source: Int J Mol Sci. 2022 May 26;23(11):6005. doi: 10.3390/ijms23116005 (PMC9181372; doi:10.3390/ijms23116005)
Supplement: Supplementary file 1 [file ijms-23-06005-s001.zip › ijms-1744280-supplementary.pdf]

Supplementary file 1: Tables

Table S1. Fold changes of gene expression in pleura and lung of mice exposed to Mitsui-7 (Mit-7) or NM-401. Statistical analysis was performed in R using means of linear regression, adjusted for total exposure time and sex (*n* = 19). High dose (HD), low dose (LD).

**Pleura**												
	Mit-7 LD			Mit-7 HD			NM-401 LD			NM-401 HD		
	Mean	SD	p-value	Mean	SD	p-value	Mean	SD	p-value	Mean	SD	p-value
Bcl-2	0.339	0.079	5.50E-04	0.422	0.099	8.00E-03	0.541	0.122	3.60E-02	0.809	0.185	4.30E-01
Ccl11	0.859	0.102	3.20E-01	0.717	0.090	5.00E-02	1.024	0.132	9.70E-01	0.983	0.122	8.40E-01
Ccl12	5.787	0.990	3.40E-10	18.238	3.481	5.10E-17	10.765	1.654	4.70E-16	17.354	2.996	6.50E-18
Ccl3	4.582	0.864	3.10E-07	5.828	1.029	3.50E-09	4.819	0.745	4.80E-09	10.448	1.836	2.60E-13
Cdh1	1.013	0.264	9.10E-01	0.994	0.273	8.70E-01	0.853	0.190	5.30E-01	1.216	0.288	6.70E-01
Col1a2	0.544	0.228	1.70E-01	1.265	0.502	8.40E-01	3.352	1.299	3.20E-02	1.771	0.602	3.40E-01
Cxcl2	3.646	0.718	9.30E-06	5.700	1.042	5.30E-09	5.105	0.799	1.60E-09	6.990	1.228	1.00E-10
Il1a	1.285	0.327	6.40E-01	1.202	0.324	8.00E-01	2.124	0.486	4.60E-02	1.521	0.366	3.40E-01
Il1b	1.673	0.595	3.80E-01	1.635	0.635	4.50E-01	2.890	0.840	2.70E-02	3.523	1.118	1.40E-02
Mmp13	0.940	0.210	6.20E-01	1.462	0.381	4.50E-01	1.637	0.349	2.10E-01	3.230	0.763	2.50E-03
Mmp2	1.124	0.124	4.80E-01	2.168	0.263	1.30E-05	1.967	0.202	1.60E-05	2.026	0.212	1.60E-05
Mmp8	2.986	0.592	7.90E-04	2.943	0.669	2.40E-03	3.071	0.581	3.30E-04	2.956	0.558	7.60E-04
Mmp9	1.071	0.304	9.20E-01	0.651	0.196	1.80E-01	1.364	0.379	5.80E-01	1.158	0.296	8.90E-01
Timp1	5.755	2.208	2.30E-03	8.203	3.191	3.40E-04	8.593	3.023	6.60E-05	14.397	4.521	1.00E-06
Timp4	0.154	0.069	7.90E-04	0.278	0.153	2.40E-02	0.329	0.119	1.80E-02	0.271	0.107	9.80E-03

**Lung**												
	Mit-7 LD			Mit-7 HD			NM-401 LD			NM-401 HD		
	Mean	SD	p-value	Mean	SD	p-value	Mean	SD	p-value	Mean	SD	p-value
Bcl-2	0.627	0.045	1.10E-05	0.870	0.066	1.80E-01	0.712	0.050	6.30E-04	0.835	0.056	6.50E-02
Ccl11	2.894	0.390	3.80E-05	2.419	0.298	5.90E-04	2.302	0.235	3.40E-04	2.344	0.263	6.90E-04
Ccl12	2.122	0.338	1.20E-03	4.122	0.694	4.30E-08	2.447	0.367	6.70E-05	3.191	0.474	7.70E-07
Ccl3	1.670	0.214	4.00E-02	1.782	0.262	2.70E-02	1.160	0.131	1.00E+00	1.786	0.203	1.00E-02
Cdh1	0.997	0.066	3.00E-01	0.961	0.066	1.60E-01	0.859	0.051	4.80E-03	0.917	0.056	4.70E-02
Cdkn2a	3.838	0.660	3.00E-06	4.064	0.698	1.10E-06	4.007	0.574	1.10E-07	4.632	0.767	8.50E-08
Col1a2	3.522	0.426	3.70E-11	5.779	0.744	1.20E-15	4.482	0.514	6.70E-15	6.012	0.658	6.00E-18
Cxcl2	2.065	0.284	3.50E-04	2.104	0.280	2.20E-04	1.489	0.174	2.80E-02	2.273	0.285	3.50E-05
Il10	4.508	0.936	8.30E-05	3.913	0.737	2.00E-04	4.294	0.803	6.50E-05	3.521	0.669	5.90E-04
Il1a	0.448	0.052	4.60E-06	0.515	0.063	1.90E-04	0.588	0.065	1.00E-03	0.413	0.045	3.40E-07
Il1b	1.061	0.178	9.70E-01	2.356	0.427	1.50E-03	1.327	0.210	3.20E-01	1.653	0.262	5.40E-02
Mmp13	0.987	0.148	8.80E-01	1.847	0.259	1.30E-03	0.596	0.084	1.40E-02	1.231	0.154	1.70E-01
Mmp2	2.110	0.155	1.70E-06	3.115	0.246	2.30E-12	2.423	0.174	3.40E-09	2.672	0.200	4.00E-10
Mmp8	1.807	0.347	3.80E-02	2.135	0.449	1.20E-02	1.571	0.267	8.50E-02	2.011	0.355	1.20E-02
Mmp9	0.952	0.140	7.80E-01	1.623	0.251	2.80E-02	1.140	0.164	5.40E-01	1.462	0.202	6.40E-02
Timp1	3.023	0.500	7.10E-06	4.455	0.721	5.80E-09	3.800	0.554	1.10E-08	3.666	0.528	3.80E-08
Timp4	1.101	0.135	6.60E-01	1.253	0.162	2.50E-01	1.237	0.143	2.30E-01	1.069	0.123	7.80E-01

Table S2. Fold change values of exposed HBEC-3KT cells. Complete list of gene expression presented as mean of fold change of exposed cells after 4 (W4), 8 (W8) and 13 (W13) weeks of exposure to NM-401 nanomaterials to the controls (*n* = 2). High dose (HD), low dose (LD).

	W4_LD		W4_HD		W8_LD		W8_HD		W13_LD		W13_HD	
	**Mean**	**SD**	**Mean**	**SD**	**Mean**	**SD**	**Mean**	**SD**	**Mean**	**SD**	**Mean**	**SD**
CASP3	1.656	0.025	1.796	0.256	1.640	0.084	1.816	0.069	1.223	0.025	1.256	0.074
CYCS	0.564	0.020	0.670	0.179	0.367	0.092	0.426	0.082	0.352	0.055	0.322	0.013
DIABLO	1.213	0.030	1.261	0.022	1.315	0.024	1.549	0.066	0.903	0.050	0.969	0.010
FADD	1.202	0.420	1.252	0.246	1.196	0.233	1.198	0.097	0.872	0.428	0.780	0.279
FAS	1.076	0.197	1.125	0.191	1.735	0.376	2.157	0.726	1.184	0.430	1.273	0.248
DRAM1	9.516	1.356	12.001	1.109	8.968	2.483	12.486	2.092	5.183	1.011	5.127	0.254
BCL-2	1.229	0.246	1.341	0.551	1.114	0.130	1.509	0.243	0.755	0.053	0.677	0.100
AKT1	1.864	0.065	1.992	0.418	1.415	0.104	1.427	0.172	1.280	0.041	1.149	0.123
NPC1	2.130	0.031	2.549	0.279	1.853	0.041	1.956	0.182	1.427	0.195	1.310	0.058
SKI	1.352	0.096	1.449	0.158	1.140	0.145	1.222	0.233	1.123	0.112	1.052	0.153
BAX	2.797	0.282	2.980	0.316	3.201	0.167	3.466	0.702	3.279	0.089	3.264	0.248
ING1	1.878	0.027	2.200	0.145	1.896	0.098	2.074	0.267	1.448	0.095	1.338	0.162
TP53	1.945	0.155	2.090	0.646	1.391	0.109	1.360	0.092	1.270	0.034	1.092	0.039
TNF	10.986	1.400	18.453	1.190	6.599	2.240	8.880	0.019	6.763	1.871	9.128	4.308
MYC	0.988	0.157	1.093	0.113	1.004	0.018	1.000	0.004	0.575	0.108	0.620	0.046
HDAC4	1.427	0.039	1.342	0.049	1.597	0.395	1.102	0.101	0.734	0.040	0.654	0.010
NEK2	0.553	0.070	0.793	0.335	0.791	0.408	0.757	0.006	0.340	0.104	0.394	0.280
CDKN2A	3.066	0.406	3.390	0.127	8.077	0.425	9.491	0.810	8.423	0.521	9.372	2.004
CDKN1B	1.850	0.069	2.015	0.246	1.559	0.096	1.681	0.020	1.408	0.064	1.420	0.115
CDKN1A	1.959	0.249	2.064	0.021	2.993	0.248	3.517	0.142	2.473	0.222	3.019	0.266
ATM	1.193	0.086	1.097	0.022	0.953	0.125	1.091	0.059	1.072	0.045	0.934	0.071
ATR	1.564	0.049	1.498	0.035	1.195	0.025	1.236	0.054	0.779	0.078	0.791	0.043
CHEK1	1.706	0.051	2.129	0.740	1.462	0.016	1.693	0.312	0.912	0.035	0.790	0.018
CHEK2	1.430	0.104	1.558	0.402	1.617	0.119	1.666	0.024	1.033	0.087	0.920	0.064
RAD1	1.878	0.169	2.131	0.207	1.984	0.149	2.211	0.047	0.979	0.005	1.112	0.025
RAD17	0.814	0.025	0.765	0.057	0.875	0.056	0.958	0.105	0.577	0.108	0.669	0.057
DNMT1	2.254	0.309	2.647	1.017	2.012	0.410	2.150	0.514	1.333	0.013	1.082	0.130
DNMT3A	1.605	0.045	1.619	0.065	1.373	0.125	1.387	0.045	1.470	0.203	1.370	0.284
DNMT3B	0.534	0.095	0.706	0.030	0.747	0.175	0.902	0.302	0.515	0.067	0.501	0.015
ALKBH1	1.763	0.076	2.129	0.124	1.759	0.028	2.004	0.383	1.017	0.167	1.023	0.213
ALKBH5	1.472	0.083	1.543	0.278	1.095	0.014	1.176	0.278	0.894	0.003	0.814	0.016
ERCC2	2.409	0.016	2.883	0.749	1.489	0.272	1.426	0.290	1.593	0.039	1.280	0.018
NEIL3	0.989	0.140	1.579	0.958	0.732	0.138	0.701	0.246	0.401	0.056	0.317	0.109
XPA	0.566	0.031	0.683	0.067	0.632	0.209	0.706	0.282	0.370	0.092	0.419	0.086
OGG1	0.909	0.099	0.936	0.169	0.953	0.083	0.973	0.032	0.658	0.101	0.690	0.119
STAT6	0.848	0.081	0.837	0.042	0.845	0.034	0.904	0.003	0.740	0.073	0.834	0.133
NFE2L2	0.642	0.150	0.649	0.118	0.945	0.378	1.099	0.426	0.563	0.234	0.658	0.226
CAT	0.618	0.029	0.746	0.121	0.986	0.040	1.044	0.045	0.834	0.031	0.806	0.012
NOX1	0.995	0.120	0.956	0.238	0.865	0.031	0.995	0.073	0.779	0.058	0.848	0.013
SOD2	11.641	0.548	12.359	0.710	12.690	0.160	14.439	0.960	6.459	1.533	7.002	1.085
IL8	2430.368	118.413	4027.346	823.579	1638.320	579.911	2255.515	374.169	172.115	23.580	183.506	51.592
TERC	1.189	0.548	0.952	0.179	0.291	0.020	0.270	0.044	0.296	0.030	0.316	0.058

TERT	5.083	2.438	3.978	0.993	5.495	0.946	4.749	0.723	7.858	1.022	4.770	0.358
CDH1	0.777	0.116	2.053	0.205	1.030	0.084	1.038	0.145	0.977	0.219	0.726	0.219
CXCL2	30.350	2.254	45.743	21.130	22.427	5.791	30.354	1.655	3.002	0.214	5.999	3.023
MMP2	1.783	0.000	10.823	3.423	2.887	0.679	4.848	4.381	6.803	3.770	10.820	6.620
CCL3	3.954	1.037	4.822	1.689	2.048	1.001	10.529	9.193	1.236	0.258	9.646	10.942

Supplementary file 1: Figures

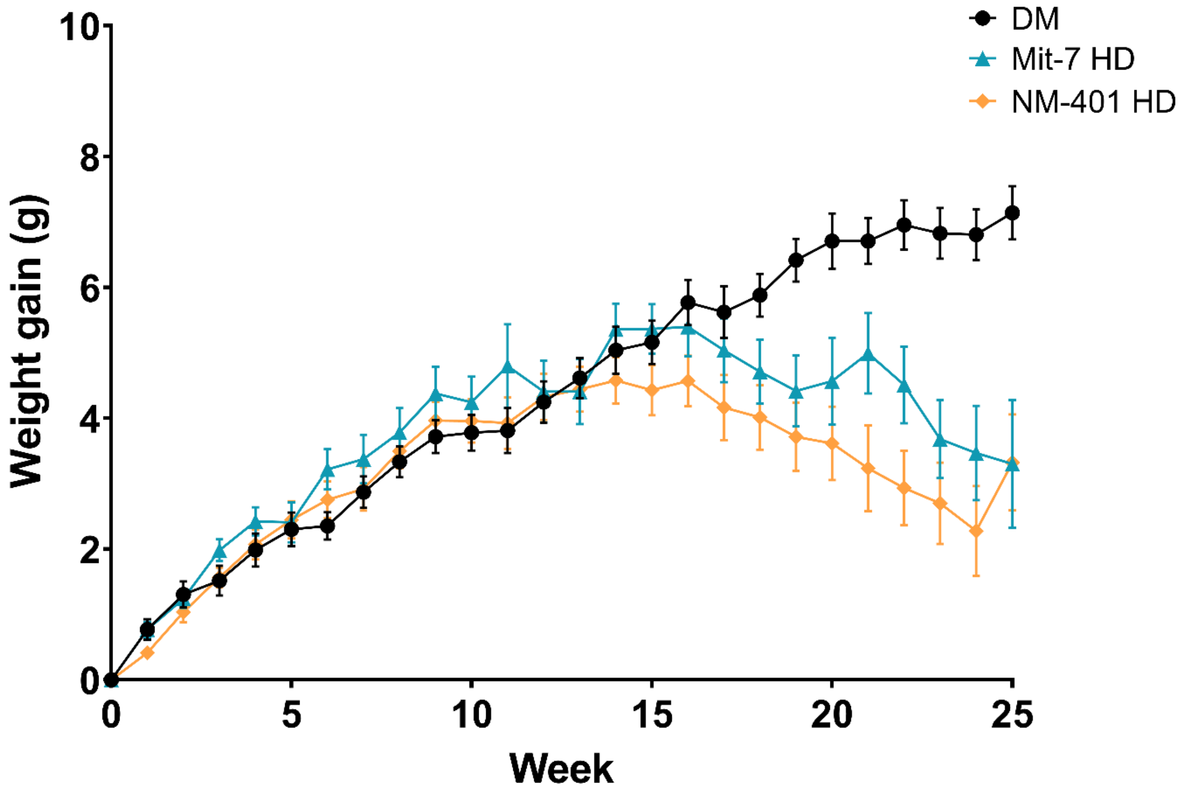

Figure S1. Body weight gain of animals after intrapleural injection of MWCNT. Body weight normalized to each animal at time 0**.** High dose (HD), Mitsui-7 (Mit-7). Values are mean ± SEM (*n* = 19).

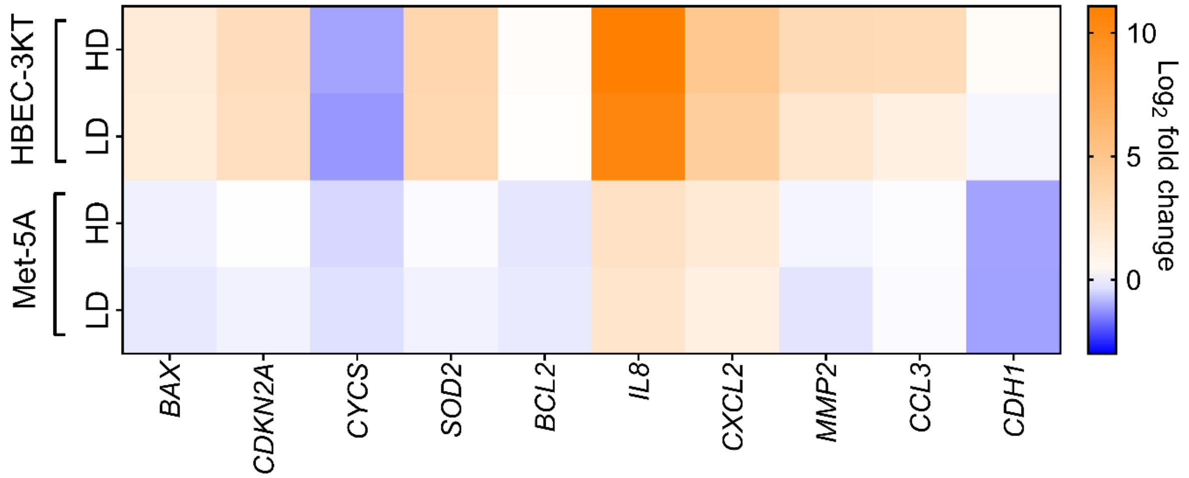

Figure S2. Heatmap illustrating selected genes involved in fibrosis and inflammation**.** Selected genes were run following NM-401 exposure in HBEC-3KT and Met-5A cells. Data presented as mean values of merged data from all weeks for low dose (LD): 0.96 µg/cm^2 and high dose (HD): 1.92 µg/cm^2 (*n* = 6).

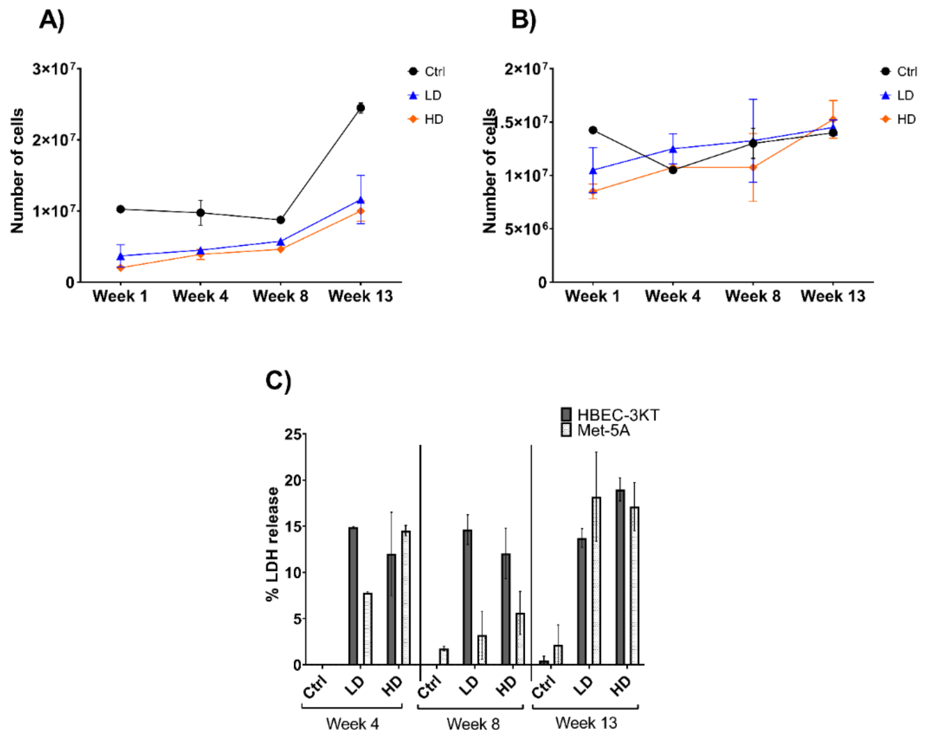

Figure S3. Cell proliferation and LDH release of exposed cells to NM-401. Cell count for HBEC-3KT cells (A) and Met-5A cells (B) for control and exposed cells assessed by trypan blue assay presented after 1, 4, 8 and 13 weeks of exposure. c) LDH assay was assessed after 4, 8 and 13 weeks of exposure. Control (Ctrl), low dose (LD): 0.96 µg/cm^2 and high dose (HD): 1.92 µg/cm^2. Data indicates mean±SEM (*n* = 2).

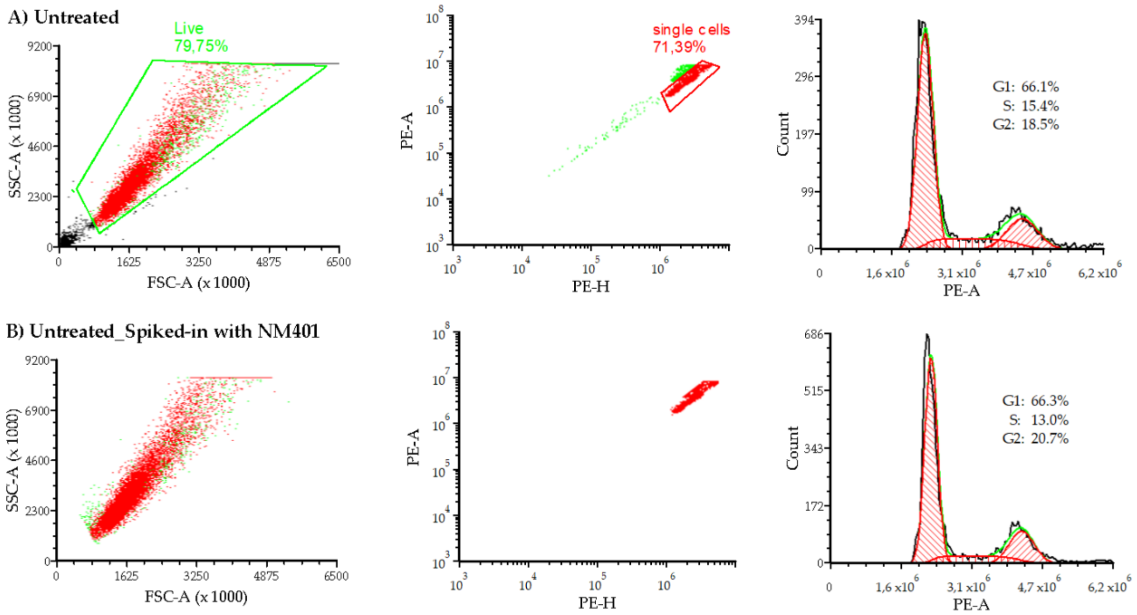

Figure S4. Assessment NM-401 nanoparticle interference with cell cycle analysis using flow cytometry. Representative scatter plots and histogram of untreated cells (A) and untreated cells spiked with high-dose of NM-401 (B).

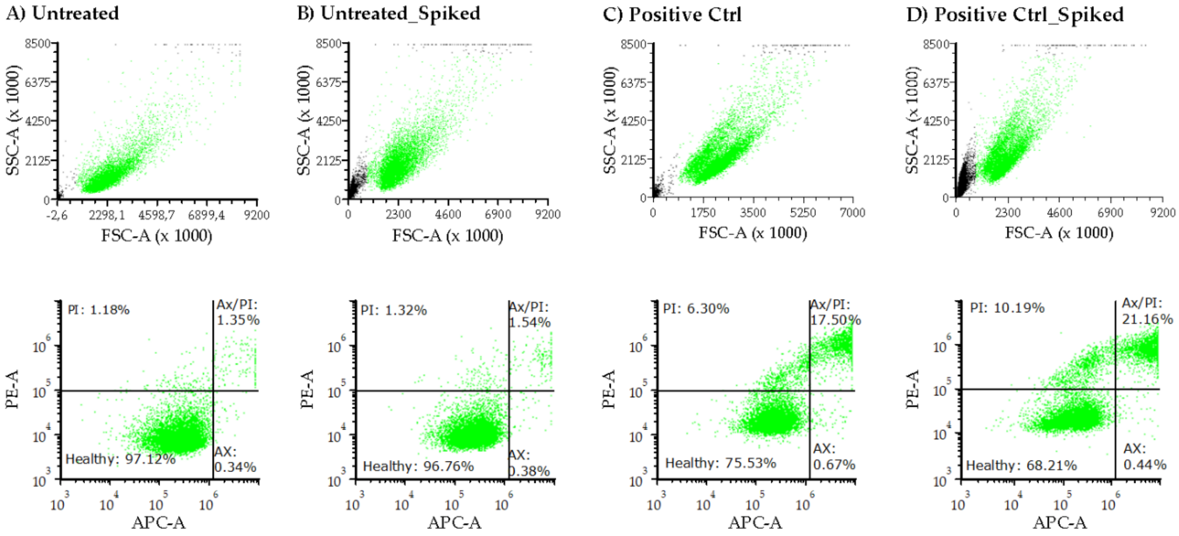

Figure S5. Assessment NM-401 nanoparticle interference with the analysis of apoptosis by flow cytometry. Representative scatter plots of untreated cells (A), untreated cells spiked with high-dose of NM-401 (B), positive control (Ctrl) i.e., cells heat shocked at 56˚C for 5 min (C) and positive control spiked with high-dose (HD = 1.92 µg/cm^2) of NM-401 (D). PI: propidium iodide and AX: Annexin-V.
